# 1-(2-Chloro­benz­yl)-3,5-dimethyl-2,6-diphenyl­piperidine

**DOI:** 10.1107/S1600536812029212

**Published:** 2012-06-30

**Authors:** Chennan Ramalingan, Seik Weng Ng, Edward R. T. Tiekink

**Affiliations:** a Centre for Nanotechnology, Department of Chemistry, Kalasalingam University, Krishnankoil - 626 126, Tamilnadu, India; b Department of Chemistry, University of Malaya, 50603 Kuala Lumpur, Malaysia; c Chemistry Department and Faculty of Science, King Abdulaziz University, PO Box 80203 Jeddah, Saudi Arabia

## Abstract

Two independent mol­ecules (*A* and *B*) comprise the asymmetric unit of the title compound, C_26_H_28_ClN, with the inverted form of *B* almost superimposable upon *A*. Each piperidine ring has a chair conformation and the chloro substituent is *anti* to the piperidine N atom. Each of two aromatic rings, the benzyl residue and one methyl group substituents occupies an equatorial position, and the second methyl substituent occupies an axial position. The dihedral angle formed between the chloro­benzene ring and the flanking phenyl rings in mol­ecule *A* are 84.24 (9) and 24.85 (8)°; the equivalent angles for mol­ecule *B* are 79.97 (9) and 28.33 (9)°. In the crystal, the *A* and *B* mol­ecules are connected by C—H⋯Cl and C—H⋯π inter­actions, forming a supra­molecular chain along [101].

## Related literature

For the biological activity of piperidine derivatives, see: Ramalingan *et al.* (2004 ▶); Ramachandran *et al.* (2011 ▶). For a related structure, see: Ramalingan *et al.* (2012 ▶). For additional conformational analysis, see: Spek (2009 ▶).
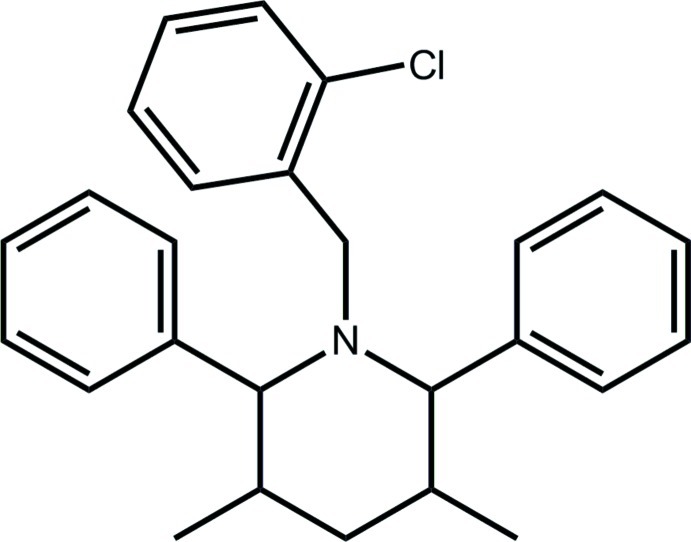


## Experimental

### 

#### Crystal data

C_26_H_28_ClN
*M* *_r_* = 389.94
Monoclinic, 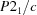

*a* = 13.4940 (6) Å
*b* = 17.3005 (6) Å
*c* = 18.5078 (6) Å
β = 100.892 (4)°
*V* = 4242.9 (3) Å^3^
*Z* = 8
Mo *K*α radiation
μ = 0.19 mm^−1^
*T* = 100 K
0.30 × 0.20 × 0.05 mm

#### Data collection

Agilent SuperNova Dual diffractometer with an Atlas detector
Absorption correction: multi-scan (*CrysAlis PRO*; Agilent, 2012 ▶) *T*_min_ = 0.718, *T*_max_ = 1.000
28999 measured reflections
9798 independent reflections
7163 reflections with *I* > 2σ(*I*)
*R*_int_ = 0.044

#### Refinement

*R*[*F* ^2^ > 2σ(*F* ^2^)] = 0.050
*wR*(*F* ^2^) = 0.125
*S* = 1.04
9798 reflections
505 parameters
H-atom parameters constrained
Δρ_max_ = 0.40 e Å^−3^
Δρ_min_ = −0.34 e Å^−3^

### 

Data collection: *CrysAlis PRO* (Agilent, 2012 ▶); cell refinement: *CrysAlis PRO*; data reduction: *CrysAlis PRO*; program(s) used to solve structure: *SHELXS97* (Sheldrick, 2008 ▶); program(s) used to refine structure: *SHELXL97* (Sheldrick, 2008 ▶); molecular graphics: *ORTEP-3 for Windows* (Farrugia, 1997 ▶), *DIAMOND* (Brandenburg, 2006 ▶) and *QMol* (Gans & Shalloway, 2001 ▶); software used to prepare material for publication: *publCIF* (Westrip, 2010 ▶).

## Supplementary Material

Crystal structure: contains datablock(s) global, I. DOI: 10.1107/S1600536812029212/hb6873sup1.cif

Structure factors: contains datablock(s) I. DOI: 10.1107/S1600536812029212/hb6873Isup2.hkl

Supplementary material file. DOI: 10.1107/S1600536812029212/hb6873Isup3.cml

Additional supplementary materials:  crystallographic information; 3D view; checkCIF report

## Figures and Tables

**Table 1:** Hydrogen-bond geometry (Å, °) *Cg*1 is the centroid of the C35–C40 ring.

*D*—H⋯*A*	*D*—H	H⋯*A*	*D*⋯*A*	*D*—H⋯*A*
C29—H29⋯Cl1	0.95	2.73	3.678 (2)	174
C24—H24⋯*Cg*1^i^	0.95	2.95	3.680 (2)	135

Symmetry code: (i) 
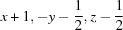
.

**Fig. 1.**
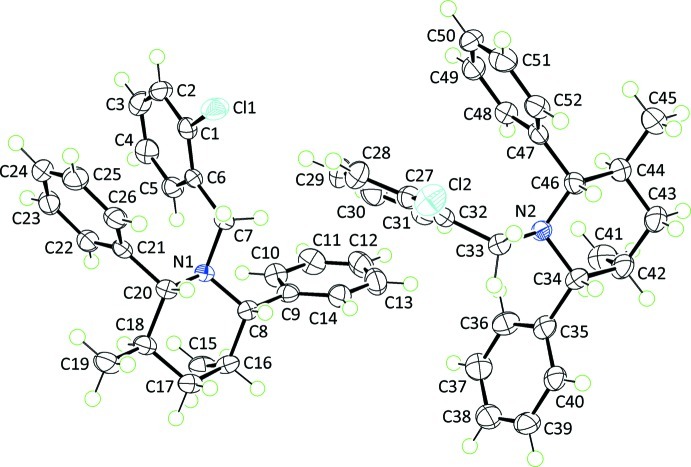
The molecular structure of (I) showing displacement ellipsoids at the 70% probability level.

**Fig. 2.**
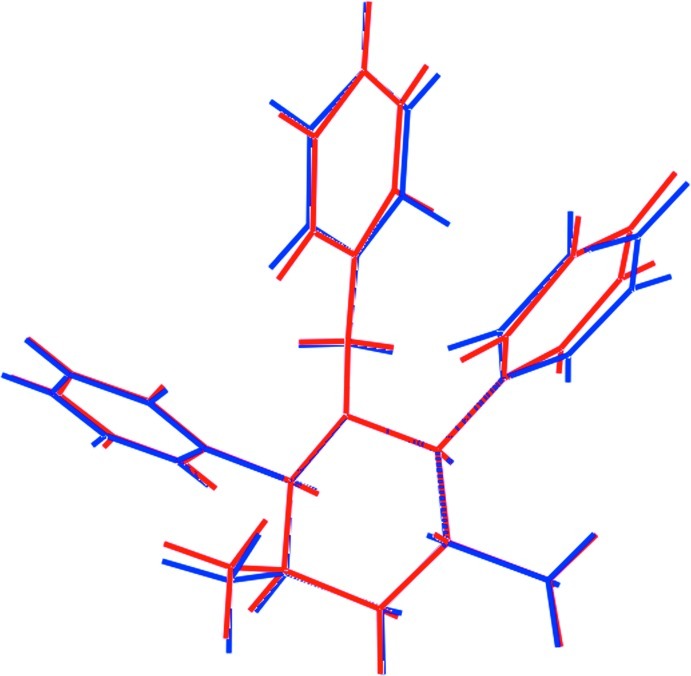
Superimposition of the two independent molecules in (I); the N2-containing molecule has been inverted. The ring-C,N,C-sequences have been superimposed, and the N1- and N2-containing molecules are shown as red and blue images, respectively.

**Fig. 3.**
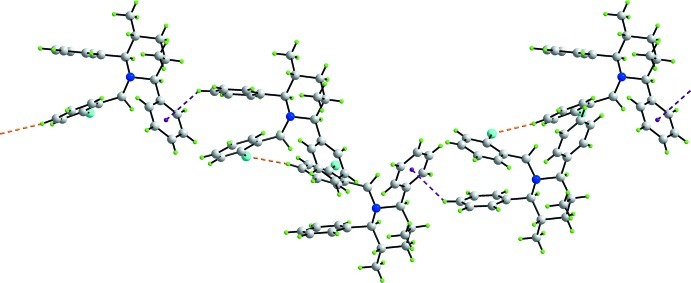
A view of the supramolecular chain in (I) sustained by C—H···Cl and C—H···π interactions, shown as orange and purple dashed lines, respectively.

**Fig. 4.**
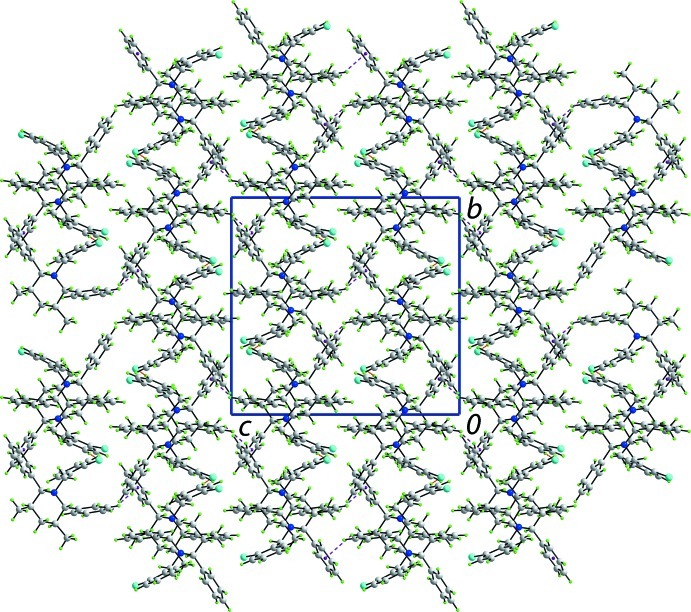
A view in projection down the a axis of the unit-cell contents for (I). The C—H···Cl and C—H···π interactions are shown as orange and purple dashed lines, respectively.

## supplementary crystallographic information

### Comment

The crystal structure determination of the title compound was undertaken in order to establish conformational details for a molecule designed and synthesized for the evaluation of its biological properties. The motivation for the biological trial arises as piperidine derivatives are an important class of heterocyclic compounds with potent pharmacological/biological activities (Ramalingan *et al.*, 2004; Ramachandran *et al.*, 2011).

Two independent molecules comprise the asymmetric unit of (I), Fig. 1. The inverted molecule of the N2-containing molecule is virtually super-imposable upon that of the N1-containing molecule, Fig. 2. The r.m.s. bond and angle fits are 0.0045 Å and 0.617°, respectively (Spek, 2009). Each piperidine ring has a chair conformation and the two aromatic rings, the benzyl residue and one methyl substituent occupy equatorial positions, as found in a related structure lacking one C-bound methyl group (Ramalingan *et al.*, 2012), with the additional methyl substituent occupying an axial position. The dihedral angle formed between the C1–C6 chlorobenzene ring and the flanking C9–C14 and C21–C26 phenyl rings are 84.24 (9) and 24.85 (8)°, respectively; the dihedral angle between the phenyl rings is 62.03 (9)°. The comparable values found for the second independent molecule are 79.97 (9), 28.33 (9) and 54.39 (8)°, respectively. The chloro substituent is *anti* to the piperidine-N atom in each independent molecule.

In the crystal, the independent molecules are connected to each other by C—H···Cl and C—H···π interactions, Table 1, to form a supramolecular chain along [101], Fig. 3. These assemble into the three-dimensional architecture without specific intermolecular interactions between them, Fig. 4.

### Experimental

A starting material, 3,5-dimethyl-2,6-diphenylpiperidine, was synthesized from benzaldehyde, 2-butanone and ammonium acetate through a Mannich-type reaction (for a typical synthesis, see Ramalingan *et al.* (2004)) followed by standard Wolff-Kishner reduction using hydrazine hydrate in diethylene glycol. The title compound was then synthesized as follows. To a DMF solution (15 ml) of 3,5-dimethyl-2,6-diphenylpiperidine (1.33 g, 0.005 mol) was added potassium *tert*-butoxide (0.67 g, 0.006 mol). The mixture was stirred for 30 minutes and 2-chlorobenzyl bromide (0.78 ml, 0.006 mol) was added drop-wise. Stirring was continued overnight before aqueous work-up. Extraction with diethyl ether followed by column chromatography separation using *n*-hexane/ethyl acetate (100:4) as an eluent eventually provided the pure title compound as a white solid. Re-crystallization was performed by slow evaporation of its ethanolic solution which afforded colourless prisms. *M*.pt: 357–358 K. Yield: 79%.

### Refinement

Carbon-bound H-atoms were placed in calculated positions [C—H = 0.95–0.99 Å, *U*_iso_(H) = 1.2–1.5*U*_eq_(C)] and were included in the refinement in the riding model approximation. Owing to poor agreement, a reflection, *i.e*. (0 0 2), was omitted from the final refinement.

### Crystal data

**Table:**

C_26_H_28_ClN	*F*(000) = 1664
*M**_r_* = 389.94	*D*_x_ = 1.221 Mg m^−^^3^
Monoclinic, *P*2_1_/*c*	Mo *K*α radiation, λ = 0.71073 Å
Hall symbol: -P 2ybc	Cell parameters from 7626 reflections
*a* = 13.4940 (6) Å	θ = 2.2–27.5°
*b* = 17.3005 (6) Å	µ = 0.19 mm^−^^1^
*c* = 18.5078 (6) Å	*T* = 100 K
β = 100.892 (4)°	Prism, colourless
*V* = 4242.9 (3) Å^3^	0.30 × 0.20 × 0.05 mm
*Z* = 8	

### Data collection

**Table:**

Agilent SuperNova Dual diffractometer with an Atlas detector	9798 independent reflections
Radiation source: SuperNova (Mo) X-ray Source	7163 reflections with *I* > 2σ(*I*)
Mirror monochromator	*R*_int_ = 0.044
Detector resolution: 10.4041 pixels mm^-1^	θ_max_ = 27.6°, θ_min_ = 2.4°
ω scan	*h* = −17→12
Absorption correction: multi-scan (*CrysAlis PRO*; Agilent, 2012)	*k* = −21→22
*T*_min_ = 0.718, *T*_max_ = 1.000	*l* = −24→24
28999 measured reflections	

### Refinement

**Table:**

Refinement on *F*^2^	Primary atom site location: structure-invariant direct methods
Least-squares matrix: full	Secondary atom site location: difference Fourier map
*R*[*F*^2^ > 2σ(*F*^2^)] = 0.050	Hydrogen site location: inferred from neighbouring sites
*wR*(*F*^2^) = 0.125	H-atom parameters constrained
*S* = 1.04	*w* = 1/[σ^2^(*F*_o_^2^) + (0.0462*P*)^2^ + 1.548*P*] where *P* = (*F*_o_^2^ + 2*F*_c_^2^)/3
9798 reflections	(Δ/σ)_max_ = 0.001
505 parameters	Δρ_max_ = 0.40 e Å^−^^3^
0 restraints	Δρ_min_ = −0.34 e Å^−^^3^

### Special details

**Table:**

Geometry. All e.s.d.'s (except the e.s.d. in the dihedral angle between two l.s. planes) are estimated using the full covariance matrix. The cell e.s.d.'s are taken into account individually in the estimation of e.s.d.'s in distances, angles and torsion angles; correlations between e.s.d.'s in cell parameters are only used when they are defined by crystal symmetry. An approximate (isotropic) treatment of cell e.s.d.'s is used for estimating e.s.d.'s involving l.s. planes.
Refinement. Refinement of *F*^2^ against ALL reflections. The weighted *R*-factor *wR* and goodness of fit *S* are based on *F*^2^, conventional *R*-factors *R* are based on *F*, with *F* set to zero for negative *F*^2^. The threshold expression of *F*^2^ > σ(*F*^2^) is used only for calculating *R*-factors(gt) *etc*. and is not relevant to the choice of reflections for refinement. *R*-factors based on *F*^2^ are statistically about twice as large as those based on *F*, and *R*- factors based on ALL data will be even larger.

### Fractional atomic coordinates and isotropic or equivalent isotropic displacement parameters (Å^2^)

**Table:**

	*x*	*y*	*z*	*U*_iso_*/*U*_eq_	
Cl1	0.57702 (4)	0.21027 (3)	0.41914 (3)	0.03073 (13)	
Cl2	0.05909 (4)	0.15772 (3)	0.44476 (2)	0.03019 (13)	
N1	0.47668 (11)	0.36054 (8)	0.22226 (8)	0.0175 (3)	
N2	−0.01535 (11)	0.01975 (8)	0.23992 (8)	0.0170 (3)	
C1	0.65192 (14)	0.23531 (10)	0.35521 (10)	0.0207 (4)	
C2	0.75323 (15)	0.21679 (10)	0.37297 (11)	0.0262 (4)	
H2	0.7794	0.1919	0.4183	0.031*	
C3	0.81625 (15)	0.23450 (11)	0.32482 (11)	0.0275 (4)	
H3	0.8863	0.2234	0.3373	0.033*	
C4	0.77627 (15)	0.26874 (11)	0.25795 (11)	0.0264 (4)	
H4	0.8186	0.2798	0.2237	0.032*	
C5	0.67491 (14)	0.28676 (10)	0.24120 (10)	0.0224 (4)	
H5	0.6488	0.3104	0.1952	0.027*	
C6	0.60939 (13)	0.27155 (9)	0.28941 (9)	0.0181 (4)	
C7	0.49834 (13)	0.29035 (10)	0.26858 (9)	0.0192 (4)	
H7A	0.4629	0.2458	0.2417	0.023*	
H7B	0.4709	0.2976	0.3141	0.023*	
C8	0.38071 (13)	0.35121 (10)	0.16845 (9)	0.0188 (4)	
H8	0.3252	0.3467	0.1972	0.023*	
C9	0.37872 (14)	0.27846 (10)	0.12260 (9)	0.0200 (4)	
C10	0.45825 (15)	0.25644 (11)	0.08894 (10)	0.0252 (4)	
H10	0.5174	0.2874	0.0951	0.030*	
C11	0.45250 (17)	0.19001 (11)	0.04664 (11)	0.0315 (5)	
H11	0.5075	0.1760	0.0241	0.038*	
C12	0.36747 (17)	0.14409 (11)	0.03707 (11)	0.0331 (5)	
H12	0.3638	0.0985	0.0080	0.040*	
C13	0.28759 (17)	0.16479 (11)	0.07005 (11)	0.0318 (5)	
H13	0.2287	0.1335	0.0637	0.038*	
C14	0.29341 (15)	0.23138 (11)	0.11253 (10)	0.0249 (4)	
H14	0.2383	0.2451	0.1351	0.030*	
C15	0.43715 (15)	0.43839 (11)	0.07032 (10)	0.0279 (4)	
H15A	0.4190	0.4852	0.0410	0.042*	
H15B	0.4381	0.3941	0.0374	0.042*	
H15C	0.5041	0.4449	0.1012	0.042*	
C16	0.35919 (14)	0.42411 (10)	0.11947 (10)	0.0225 (4)	
H16	0.2921	0.4168	0.0865	0.027*	
C17	0.35038 (14)	0.49333 (10)	0.16897 (10)	0.0239 (4)	
H17A	0.3386	0.5407	0.1385	0.029*	
H17B	0.2916	0.4859	0.1932	0.029*	
C18	0.44526 (15)	0.50369 (10)	0.22750 (10)	0.0224 (4)	
H18	0.5028	0.5163	0.2026	0.027*	
C19	0.43245 (17)	0.56965 (11)	0.27974 (11)	0.0329 (5)	
H19A	0.4188	0.6178	0.2517	0.049*	
H19B	0.4944	0.5754	0.3167	0.049*	
H19C	0.3759	0.5581	0.3043	0.049*	
C20	0.46965 (14)	0.42816 (10)	0.27099 (9)	0.0180 (4)	
H20	0.4126	0.4179	0.2972	0.022*	
C21	0.56471 (14)	0.43588 (9)	0.32922 (9)	0.0178 (4)	
C22	0.65728 (14)	0.45263 (10)	0.30990 (10)	0.0212 (4)	
H22	0.6605	0.4618	0.2598	0.025*	
C23	0.74484 (15)	0.45603 (10)	0.36293 (10)	0.0245 (4)	
H23	0.8075	0.4672	0.3490	0.029*	
C24	0.74092 (15)	0.44318 (10)	0.43634 (10)	0.0263 (4)	
H24	0.8010	0.4442	0.4726	0.032*	
C25	0.64913 (15)	0.42898 (11)	0.45631 (10)	0.0265 (4)	
H25	0.6457	0.4217	0.5067	0.032*	
C26	0.56187 (15)	0.42539 (10)	0.40311 (10)	0.0219 (4)	
H26	0.4991	0.4156	0.4175	0.026*	
C27	0.14146 (14)	0.14577 (10)	0.38294 (10)	0.0216 (4)	
C28	0.24042 (15)	0.16987 (12)	0.40676 (11)	0.0306 (5)	
H28	0.2610	0.1911	0.4546	0.037*	
C29	0.30822 (16)	0.16268 (12)	0.36024 (12)	0.0354 (5)	
H29	0.3760	0.1792	0.3758	0.043*	
C30	0.27767 (15)	0.13137 (11)	0.29056 (11)	0.0305 (5)	
H30	0.3244	0.1263	0.2584	0.037*	
C31	0.17902 (14)	0.10758 (10)	0.26834 (10)	0.0235 (4)	
H31	0.1590	0.0864	0.2205	0.028*	
C32	0.10767 (13)	0.11353 (9)	0.31353 (9)	0.0176 (4)	
C33	−0.00109 (13)	0.09102 (10)	0.28530 (9)	0.0175 (4)	
H33A	−0.0347	0.0839	0.3280	0.021*	
H33B	−0.0353	0.1343	0.2556	0.021*	
C34	−0.10840 (13)	0.02830 (10)	0.18282 (9)	0.0196 (4)	
H34	−0.1656	0.0371	0.2092	0.024*	
C35	−0.10346 (14)	0.09797 (10)	0.13410 (9)	0.0210 (4)	
C36	−0.01872 (15)	0.11497 (11)	0.10387 (10)	0.0265 (4)	
H36	0.0386	0.0821	0.1141	0.032*	
C37	−0.01681 (16)	0.17884 (11)	0.05930 (10)	0.0296 (5)	
H37	0.0417	0.1896	0.0394	0.036*	
C38	−0.09997 (16)	0.22715 (12)	0.04353 (10)	0.0311 (5)	
H38	−0.0985	0.2713	0.0132	0.037*	
C39	−0.18465 (16)	0.21079 (11)	0.07201 (10)	0.0289 (4)	
H39	−0.2424	0.2431	0.0605	0.035*	
C40	−0.18589 (14)	0.14725 (10)	0.11758 (10)	0.0230 (4)	
H40	−0.2442	0.1373	0.1379	0.028*	
C41	−0.05959 (17)	−0.06385 (12)	0.08492 (11)	0.0325 (5)	
H41A	−0.0799	−0.1120	0.0583	0.049*	
H41B	0.0086	−0.0697	0.1140	0.049*	
H41C	−0.0603	−0.0215	0.0496	0.049*	
C42	−0.13320 (15)	−0.04568 (11)	0.13610 (10)	0.0251 (4)	
H42	−0.2012	−0.0379	0.1044	0.030*	
C43	−0.14212 (15)	−0.11296 (11)	0.18682 (11)	0.0285 (4)	
H43A	−0.1549	−0.1610	0.1575	0.034*	
H43B	−0.2003	−0.1043	0.2114	0.034*	
C44	−0.04682 (15)	−0.12267 (10)	0.24478 (10)	0.0243 (4)	
H44	0.0109	−0.1333	0.2194	0.029*	
C45	−0.05674 (17)	−0.18987 (11)	0.29566 (12)	0.0356 (5)	
H45A	−0.0686	−0.2377	0.2669	0.053*	
H45B	−0.1136	−0.1805	0.3205	0.053*	
H45C	0.0055	−0.1948	0.3324	0.053*	
C46	−0.02524 (14)	−0.04680 (10)	0.28885 (10)	0.0196 (4)	
H46	−0.0847	−0.0363	0.3125	0.024*	
C47	0.06641 (13)	−0.05358 (9)	0.34988 (9)	0.0185 (4)	
C48	0.16218 (14)	−0.06415 (10)	0.33366 (10)	0.0211 (4)	
H48	0.1701	−0.0667	0.2838	0.025*	
C49	0.24588 (14)	−0.07102 (10)	0.38926 (10)	0.0240 (4)	
H49	0.3108	−0.0777	0.3774	0.029*	
C50	0.23543 (15)	−0.06826 (10)	0.46231 (10)	0.0255 (4)	
H50	0.2930	−0.0731	0.5005	0.031*	
C51	0.14104 (15)	−0.05841 (10)	0.47917 (10)	0.0262 (4)	
H51	0.1335	−0.0566	0.5291	0.031*	
C52	0.05683 (14)	−0.05107 (10)	0.42327 (10)	0.0220 (4)	
H52	−0.0079	−0.0443	0.4354	0.026*	

### Atomic displacement parameters (Å^2^)

**Table:**

	*U*^11^	*U*^22^	*U*^33^	*U*^12^	*U*^13^	*U*^23^
Cl1	0.0274 (3)	0.0376 (3)	0.0254 (2)	−0.0017 (2)	0.0003 (2)	0.0128 (2)
Cl2	0.0320 (3)	0.0374 (3)	0.0208 (2)	0.0034 (2)	0.0040 (2)	−0.0100 (2)
N1	0.0189 (8)	0.0143 (7)	0.0178 (7)	0.0011 (6)	0.0000 (6)	0.0019 (6)
N2	0.0166 (8)	0.0158 (7)	0.0177 (7)	0.0011 (6)	0.0009 (6)	−0.0017 (6)
C1	0.0246 (10)	0.0156 (8)	0.0210 (9)	−0.0019 (7)	0.0022 (7)	−0.0023 (7)
C2	0.0271 (11)	0.0206 (9)	0.0269 (10)	0.0053 (8)	−0.0053 (8)	−0.0010 (8)
C3	0.0202 (10)	0.0245 (10)	0.0354 (11)	0.0062 (8)	−0.0008 (8)	−0.0091 (9)
C4	0.0247 (11)	0.0245 (10)	0.0315 (10)	0.0034 (8)	0.0087 (8)	−0.0060 (8)
C5	0.0256 (11)	0.0185 (9)	0.0225 (9)	0.0017 (7)	0.0027 (8)	−0.0008 (7)
C6	0.0201 (10)	0.0112 (8)	0.0214 (9)	0.0006 (7)	0.0000 (7)	−0.0021 (7)
C7	0.0205 (10)	0.0156 (8)	0.0210 (9)	−0.0008 (7)	0.0026 (7)	0.0041 (7)
C8	0.0180 (9)	0.0195 (9)	0.0186 (8)	0.0010 (7)	0.0025 (7)	0.0008 (7)
C9	0.0226 (10)	0.0206 (9)	0.0159 (8)	0.0026 (7)	0.0013 (7)	0.0021 (7)
C10	0.0263 (11)	0.0258 (10)	0.0241 (9)	−0.0009 (8)	0.0064 (8)	−0.0007 (8)
C11	0.0382 (13)	0.0298 (11)	0.0290 (10)	0.0041 (9)	0.0128 (9)	−0.0030 (9)
C12	0.0483 (14)	0.0216 (10)	0.0297 (11)	−0.0008 (9)	0.0080 (10)	−0.0056 (8)
C13	0.0358 (13)	0.0255 (10)	0.0331 (11)	−0.0070 (9)	0.0042 (9)	−0.0020 (9)
C14	0.0257 (11)	0.0243 (9)	0.0246 (9)	−0.0001 (8)	0.0042 (8)	0.0007 (8)
C15	0.0306 (12)	0.0291 (10)	0.0236 (10)	0.0049 (8)	0.0041 (8)	0.0083 (8)
C16	0.0192 (10)	0.0249 (9)	0.0213 (9)	0.0045 (7)	−0.0013 (7)	0.0033 (8)
C17	0.0245 (11)	0.0208 (9)	0.0249 (9)	0.0047 (8)	0.0003 (8)	0.0041 (8)
C18	0.0266 (11)	0.0172 (9)	0.0218 (9)	0.0018 (7)	0.0008 (8)	0.0038 (7)
C19	0.0404 (13)	0.0178 (9)	0.0362 (11)	0.0071 (9)	−0.0040 (10)	−0.0001 (9)
C20	0.0198 (10)	0.0166 (8)	0.0178 (8)	0.0018 (7)	0.0044 (7)	0.0020 (7)
C21	0.0212 (10)	0.0114 (8)	0.0203 (9)	0.0017 (7)	0.0024 (7)	0.0007 (7)
C22	0.0257 (10)	0.0193 (9)	0.0186 (9)	0.0000 (7)	0.0042 (7)	0.0000 (7)
C23	0.0226 (10)	0.0202 (9)	0.0305 (10)	−0.0021 (8)	0.0042 (8)	−0.0008 (8)
C24	0.0274 (11)	0.0218 (9)	0.0255 (10)	−0.0031 (8)	−0.0056 (8)	0.0015 (8)
C25	0.0345 (12)	0.0257 (10)	0.0179 (9)	−0.0007 (8)	0.0014 (8)	0.0031 (8)
C26	0.0249 (10)	0.0186 (9)	0.0224 (9)	0.0000 (7)	0.0047 (8)	0.0038 (7)
C27	0.0235 (10)	0.0197 (9)	0.0213 (9)	0.0012 (7)	0.0031 (8)	0.0002 (7)
C28	0.0285 (12)	0.0318 (11)	0.0279 (10)	−0.0065 (9)	−0.0043 (9)	−0.0003 (9)
C29	0.0245 (12)	0.0376 (12)	0.0412 (12)	−0.0112 (9)	−0.0016 (9)	0.0052 (10)
C30	0.0250 (11)	0.0322 (11)	0.0371 (11)	−0.0028 (9)	0.0125 (9)	0.0091 (9)
C31	0.0263 (11)	0.0218 (9)	0.0228 (9)	−0.0004 (8)	0.0054 (8)	0.0036 (8)
C32	0.0199 (9)	0.0138 (8)	0.0188 (8)	0.0001 (7)	0.0027 (7)	0.0022 (7)
C33	0.0181 (9)	0.0189 (8)	0.0154 (8)	0.0018 (7)	0.0031 (7)	−0.0015 (7)
C34	0.0172 (9)	0.0220 (9)	0.0185 (8)	0.0028 (7)	0.0006 (7)	−0.0024 (7)
C35	0.0235 (10)	0.0246 (9)	0.0132 (8)	−0.0013 (8)	−0.0005 (7)	−0.0045 (7)
C36	0.0254 (11)	0.0319 (10)	0.0218 (9)	0.0046 (8)	0.0036 (8)	0.0006 (8)
C37	0.0350 (12)	0.0337 (11)	0.0204 (9)	−0.0028 (9)	0.0062 (8)	0.0013 (8)
C38	0.0367 (13)	0.0294 (10)	0.0232 (10)	−0.0034 (9)	−0.0048 (9)	0.0038 (8)
C39	0.0306 (12)	0.0252 (10)	0.0262 (10)	0.0027 (8)	−0.0069 (9)	0.0001 (8)
C40	0.0229 (10)	0.0228 (9)	0.0214 (9)	−0.0017 (8)	−0.0005 (8)	−0.0052 (8)
C41	0.0372 (13)	0.0323 (11)	0.0266 (10)	0.0041 (9)	0.0025 (9)	−0.0042 (9)
C42	0.0234 (10)	0.0242 (9)	0.0250 (9)	−0.0007 (8)	−0.0027 (8)	−0.0061 (8)
C43	0.0242 (11)	0.0241 (10)	0.0346 (11)	−0.0032 (8)	−0.0016 (9)	−0.0035 (9)
C44	0.0237 (10)	0.0190 (9)	0.0282 (10)	−0.0021 (7)	−0.0002 (8)	0.0003 (8)
C45	0.0379 (13)	0.0245 (10)	0.0407 (12)	−0.0053 (9)	−0.0016 (10)	0.0054 (9)
C46	0.0170 (9)	0.0182 (9)	0.0244 (9)	−0.0004 (7)	0.0055 (7)	0.0028 (7)
C47	0.0198 (10)	0.0135 (8)	0.0225 (9)	−0.0013 (7)	0.0042 (7)	0.0014 (7)
C48	0.0219 (10)	0.0200 (9)	0.0213 (9)	0.0001 (7)	0.0038 (7)	0.0013 (7)
C49	0.0184 (10)	0.0231 (9)	0.0306 (10)	0.0010 (7)	0.0050 (8)	0.0015 (8)
C50	0.0248 (11)	0.0215 (9)	0.0259 (10)	0.0002 (8)	−0.0060 (8)	0.0010 (8)
C51	0.0352 (12)	0.0229 (9)	0.0198 (9)	0.0015 (8)	0.0035 (8)	−0.0006 (8)
C52	0.0232 (10)	0.0193 (9)	0.0248 (9)	0.0023 (7)	0.0076 (8)	0.0029 (7)

### Geometric parameters (Å, º)

**Table:**

Cl1—C1	1.7497 (18)	C24—H24	0.9500
Cl2—C27	1.7516 (19)	C25—C26	1.386 (3)
N1—C7	1.483 (2)	C25—H25	0.9500
N1—C8	1.486 (2)	C26—H26	0.9500
N1—C20	1.491 (2)	C27—C28	1.389 (3)
N2—C33	1.484 (2)	C27—C32	1.396 (2)
N2—C46	1.486 (2)	C28—C29	1.375 (3)
N2—C34	1.488 (2)	C28—H28	0.9500
C1—C2	1.382 (3)	C29—C30	1.388 (3)
C1—C6	1.393 (2)	C29—H29	0.9500
C2—C3	1.377 (3)	C30—C31	1.380 (3)
C2—H2	0.9500	C30—H30	0.9500
C3—C4	1.386 (3)	C31—C32	1.393 (2)
C3—H3	0.9500	C31—H31	0.9500
C4—C5	1.380 (3)	C32—C33	1.513 (2)
C4—H4	0.9500	C33—H33A	0.9900
C5—C6	1.395 (2)	C33—H33B	0.9900
C5—H5	0.9500	C34—C35	1.514 (2)
C6—C7	1.510 (2)	C34—C42	1.546 (2)
C7—H7A	0.9900	C34—H34	1.0000
C7—H7B	0.9900	C35—C40	1.389 (3)
C8—C9	1.515 (2)	C35—C36	1.396 (3)
C8—C16	1.548 (2)	C36—C37	1.382 (3)
C8—H8	1.0000	C36—H36	0.9500
C9—C10	1.392 (3)	C37—C38	1.385 (3)
C9—C14	1.394 (3)	C37—H37	0.9500
C10—C11	1.384 (3)	C38—C39	1.375 (3)
C10—H10	0.9500	C38—H38	0.9500
C11—C12	1.379 (3)	C39—C40	1.388 (3)
C11—H11	0.9500	C39—H39	0.9500
C12—C13	1.383 (3)	C40—H40	0.9500
C12—H12	0.9500	C41—C42	1.529 (3)
C13—C14	1.389 (3)	C41—H41A	0.9800
C13—H13	0.9500	C41—H41B	0.9800
C14—H14	0.9500	C41—H41C	0.9800
C15—C16	1.535 (3)	C42—C43	1.514 (3)
C15—H15A	0.9800	C42—H42	1.0000
C15—H15B	0.9800	C43—C44	1.520 (3)
C15—H15C	0.9800	C43—H43A	0.9900
C16—C17	1.526 (3)	C43—H43B	0.9900
C16—H16	1.0000	C44—C45	1.518 (3)
C17—C18	1.523 (3)	C44—C46	1.544 (2)
C17—H17A	0.9900	C44—H44	1.0000
C17—H17B	0.9900	C45—H45A	0.9800
C18—C19	1.526 (3)	C45—H45B	0.9800
C18—C20	1.538 (2)	C45—H45C	0.9800
C18—H18	1.0000	C46—C47	1.514 (2)
C19—H19A	0.9800	C46—H46	1.0000
C19—H19B	0.9800	C47—C52	1.389 (2)
C19—H19C	0.9800	C47—C48	1.393 (2)
C20—C21	1.517 (2)	C48—C49	1.382 (3)
C20—H20	1.0000	C48—H48	0.9500
C21—C26	1.387 (2)	C49—C50	1.386 (3)
C21—C22	1.393 (2)	C49—H49	0.9500
C22—C23	1.387 (3)	C50—C51	1.378 (3)
C22—H22	0.9500	C50—H50	0.9500
C23—C24	1.388 (3)	C51—C52	1.391 (3)
C23—H23	0.9500	C51—H51	0.9500
C24—C25	1.380 (3)	C52—H52	0.9500
			
C7—N1—C8	110.49 (13)	C25—C26—C21	121.09 (18)
C7—N1—C20	108.71 (13)	C25—C26—H26	119.5
C8—N1—C20	110.10 (13)	C21—C26—H26	119.5
C33—N2—C46	108.41 (13)	C28—C27—C32	122.65 (17)
C33—N2—C34	108.74 (13)	C28—C27—Cl2	116.61 (14)
C46—N2—C34	110.42 (13)	C32—C27—Cl2	120.74 (14)
C2—C1—C6	122.54 (17)	C29—C28—C27	119.18 (19)
C2—C1—Cl1	116.98 (14)	C29—C28—H28	120.4
C6—C1—Cl1	120.48 (14)	C27—C28—H28	120.4
C3—C2—C1	119.90 (18)	C28—C29—C30	120.08 (19)
C3—C2—H2	120.1	C28—C29—H29	120.0
C1—C2—H2	120.1	C30—C29—H29	120.0
C2—C3—C4	119.27 (18)	C31—C30—C29	119.59 (19)
C2—C3—H3	120.4	C31—C30—H30	120.2
C4—C3—H3	120.4	C29—C30—H30	120.2
C5—C4—C3	119.95 (18)	C30—C31—C32	122.43 (18)
C5—C4—H4	120.0	C30—C31—H31	118.8
C3—C4—H4	120.0	C32—C31—H31	118.8
C4—C5—C6	122.34 (17)	C31—C32—C27	116.06 (16)
C4—C5—H5	118.8	C31—C32—C33	120.59 (16)
C6—C5—H5	118.8	C27—C32—C33	123.21 (15)
C1—C6—C5	115.97 (16)	N2—C33—C32	115.03 (14)
C1—C6—C7	123.02 (16)	N2—C33—H33A	108.5
C5—C6—C7	120.95 (16)	C32—C33—H33A	108.5
N1—C7—C6	113.67 (14)	N2—C33—H33B	108.5
N1—C7—H7A	108.8	C32—C33—H33B	108.5
C6—C7—H7A	108.8	H33A—C33—H33B	107.5
N1—C7—H7B	108.8	N2—C34—C35	112.08 (14)
C6—C7—H7B	108.8	N2—C34—C42	112.31 (14)
H7A—C7—H7B	107.7	C35—C34—C42	110.81 (14)
N1—C8—C9	112.74 (14)	N2—C34—H34	107.1
N1—C8—C16	110.31 (14)	C35—C34—H34	107.1
C9—C8—C16	111.54 (14)	C42—C34—H34	107.1
N1—C8—H8	107.3	C40—C35—C36	117.71 (17)
C9—C8—H8	107.3	C40—C35—C34	119.74 (16)
C16—C8—H8	107.3	C36—C35—C34	122.54 (17)
C10—C9—C14	117.87 (17)	C37—C36—C35	121.11 (18)
C10—C9—C8	122.98 (16)	C37—C36—H36	119.4
C14—C9—C8	119.15 (16)	C35—C36—H36	119.4
C11—C10—C9	120.97 (18)	C36—C37—C38	120.15 (19)
C11—C10—H10	119.5	C36—C37—H37	119.9
C9—C10—H10	119.5	C38—C37—H37	119.9
C12—C11—C10	120.48 (19)	C39—C38—C37	119.59 (19)
C12—C11—H11	119.8	C39—C38—H38	120.2
C10—C11—H11	119.8	C37—C38—H38	120.2
C11—C12—C13	119.54 (19)	C38—C39—C40	120.15 (19)
C11—C12—H12	120.2	C38—C39—H39	119.9
C13—C12—H12	120.2	C40—C39—H39	119.9
C12—C13—C14	119.97 (19)	C39—C40—C35	121.26 (18)
C12—C13—H13	120.0	C39—C40—H40	119.4
C14—C13—H13	120.0	C35—C40—H40	119.4
C13—C14—C9	121.17 (18)	C42—C41—H41A	109.5
C13—C14—H14	119.4	C42—C41—H41B	109.5
C9—C14—H14	119.4	H41A—C41—H41B	109.5
C16—C15—H15A	109.5	C42—C41—H41C	109.5
C16—C15—H15B	109.5	H41A—C41—H41C	109.5
H15A—C15—H15B	109.5	H41B—C41—H41C	109.5
C16—C15—H15C	109.5	C43—C42—C41	111.28 (16)
H15A—C15—H15C	109.5	C43—C42—C34	108.89 (15)
H15B—C15—H15C	109.5	C41—C42—C34	114.80 (16)
C17—C16—C15	111.67 (16)	C43—C42—H42	107.2
C17—C16—C8	108.37 (14)	C41—C42—H42	107.2
C15—C16—C8	113.85 (15)	C34—C42—H42	107.2
C17—C16—H16	107.6	C42—C43—C44	111.51 (16)
C15—C16—H16	107.6	C42—C43—H43A	109.3
C8—C16—H16	107.6	C44—C43—H43A	109.3
C18—C17—C16	111.64 (15)	C42—C43—H43B	109.3
C18—C17—H17A	109.3	C44—C43—H43B	109.3
C16—C17—H17A	109.3	H43A—C43—H43B	108.0
C18—C17—H17B	109.3	C45—C44—C43	111.13 (16)
C16—C17—H17B	109.3	C45—C44—C46	110.70 (16)
H17A—C17—H17B	108.0	C43—C44—C46	109.20 (15)
C17—C18—C19	110.97 (16)	C45—C44—H44	108.6
C17—C18—C20	109.90 (15)	C43—C44—H44	108.6
C19—C18—C20	110.02 (15)	C46—C44—H44	108.6
C17—C18—H18	108.6	C44—C45—H45A	109.5
C19—C18—H18	108.6	C44—C45—H45B	109.5
C20—C18—H18	108.6	H45A—C45—H45B	109.5
C18—C19—H19A	109.5	C44—C45—H45C	109.5
C18—C19—H19B	109.5	H45A—C45—H45C	109.5
H19A—C19—H19B	109.5	H45B—C45—H45C	109.5
C18—C19—H19C	109.5	N2—C46—C47	111.10 (14)
H19A—C19—H19C	109.5	N2—C46—C44	111.50 (14)
H19B—C19—H19C	109.5	C47—C46—C44	111.98 (14)
N1—C20—C21	110.91 (14)	N2—C46—H46	107.3
N1—C20—C18	112.45 (14)	C47—C46—H46	107.3
C21—C20—C18	111.45 (14)	C44—C46—H46	107.3
N1—C20—H20	107.2	C52—C47—C48	118.44 (17)
C21—C20—H20	107.2	C52—C47—C46	120.89 (16)
C18—C20—H20	107.2	C48—C47—C46	120.66 (15)
C26—C21—C22	118.25 (17)	C49—C48—C47	120.79 (17)
C26—C21—C20	120.76 (16)	C49—C48—H48	119.6
C22—C21—C20	120.98 (15)	C47—C48—H48	119.6
C23—C22—C21	120.83 (17)	C48—C49—C50	120.26 (18)
C23—C22—H22	119.6	C48—C49—H49	119.9
C21—C22—H22	119.6	C50—C49—H49	119.9
C22—C23—C24	120.06 (18)	C51—C50—C49	119.58 (18)
C22—C23—H23	120.0	C51—C50—H50	120.2
C24—C23—H23	120.0	C49—C50—H50	120.2
C25—C24—C23	119.54 (18)	C50—C51—C52	120.21 (17)
C25—C24—H24	120.2	C50—C51—H51	119.9
C23—C24—H24	120.2	C52—C51—H51	119.9
C24—C25—C26	120.18 (17)	C47—C52—C51	120.71 (18)
C24—C25—H25	119.9	C47—C52—H52	119.6
C26—C25—H25	119.9	C51—C52—H52	119.6
			
C6—C1—C2—C3	0.3 (3)	C32—C27—C28—C29	0.7 (3)
Cl1—C1—C2—C3	179.61 (14)	Cl2—C27—C28—C29	−179.30 (16)
C1—C2—C3—C4	−1.9 (3)	C27—C28—C29—C30	−0.3 (3)
C2—C3—C4—C5	1.9 (3)	C28—C29—C30—C31	0.0 (3)
C3—C4—C5—C6	−0.2 (3)	C29—C30—C31—C32	−0.2 (3)
C2—C1—C6—C5	1.4 (2)	C30—C31—C32—C27	0.5 (3)
Cl1—C1—C6—C5	−177.97 (13)	C30—C31—C32—C33	176.43 (17)
C2—C1—C6—C7	178.49 (16)	C28—C27—C32—C31	−0.8 (3)
Cl1—C1—C6—C7	−0.8 (2)	Cl2—C27—C32—C31	179.19 (13)
C4—C5—C6—C1	−1.4 (3)	C28—C27—C32—C33	−176.59 (17)
C4—C5—C6—C7	−178.57 (16)	Cl2—C27—C32—C33	3.4 (2)
C8—N1—C7—C6	145.16 (14)	C46—N2—C33—C32	93.22 (16)
C20—N1—C7—C6	−93.91 (17)	C34—N2—C33—C32	−146.70 (14)
C1—C6—C7—N1	149.01 (16)	C31—C32—C33—N2	41.3 (2)
C5—C6—C7—N1	−34.0 (2)	C27—C32—C33—N2	−143.10 (16)
C7—N1—C8—C9	−53.74 (18)	C33—N2—C34—C35	58.33 (17)
C20—N1—C8—C9	−173.84 (14)	C46—N2—C34—C35	177.16 (14)
C7—N1—C8—C16	−179.17 (14)	C33—N2—C34—C42	−176.17 (14)
C20—N1—C8—C16	60.73 (17)	C46—N2—C34—C42	−57.34 (18)
N1—C8—C9—C10	−46.9 (2)	N2—C34—C35—C40	−134.52 (16)
C16—C8—C9—C10	77.9 (2)	C42—C34—C35—C40	99.16 (19)
N1—C8—C9—C14	133.87 (16)	N2—C34—C35—C36	46.1 (2)
C16—C8—C9—C14	−101.37 (19)	C42—C34—C35—C36	−80.2 (2)
C14—C9—C10—C11	0.2 (3)	C40—C35—C36—C37	0.3 (3)
C8—C9—C10—C11	−179.05 (17)	C34—C35—C36—C37	179.68 (17)
C9—C10—C11—C12	−0.1 (3)	C35—C36—C37—C38	−0.3 (3)
C10—C11—C12—C13	0.0 (3)	C36—C37—C38—C39	−0.5 (3)
C11—C12—C13—C14	−0.1 (3)	C37—C38—C39—C40	1.4 (3)
C12—C13—C14—C9	0.2 (3)	C38—C39—C40—C35	−1.5 (3)
C10—C9—C14—C13	−0.3 (3)	C36—C35—C40—C39	0.6 (3)
C8—C9—C14—C13	179.02 (17)	C34—C35—C40—C39	−178.78 (16)
N1—C8—C16—C17	−60.37 (18)	N2—C34—C42—C43	56.4 (2)
C9—C8—C16—C17	173.53 (15)	C35—C34—C42—C43	−177.41 (15)
N1—C8—C16—C15	64.54 (19)	N2—C34—C42—C41	−69.1 (2)
C9—C8—C16—C15	−61.6 (2)	C35—C34—C42—C41	57.1 (2)
C15—C16—C17—C18	−68.85 (19)	C41—C42—C43—C44	71.1 (2)
C8—C16—C17—C18	57.3 (2)	C34—C42—C43—C44	−56.4 (2)
C16—C17—C18—C19	−176.09 (15)	C42—C43—C44—C45	179.61 (16)
C16—C17—C18—C20	−54.2 (2)	C42—C43—C44—C46	57.2 (2)
C7—N1—C20—C21	55.53 (17)	C33—N2—C46—C47	−57.78 (17)
C8—N1—C20—C21	176.70 (13)	C34—N2—C46—C47	−176.81 (13)
C7—N1—C20—C18	−178.92 (14)	C33—N2—C46—C44	176.55 (14)
C8—N1—C20—C18	−57.75 (18)	C34—N2—C46—C44	57.52 (18)
C17—C18—C20—N1	53.9 (2)	C45—C44—C46—N2	−179.96 (16)
C19—C18—C20—N1	176.42 (15)	C43—C44—C46—N2	−57.3 (2)
C17—C18—C20—C21	179.20 (14)	C45—C44—C46—C47	54.9 (2)
C19—C18—C20—C21	−58.3 (2)	C43—C44—C46—C47	177.52 (15)
N1—C20—C21—C26	−114.11 (17)	N2—C46—C47—C52	121.84 (17)
C18—C20—C21—C26	119.78 (17)	C44—C46—C47—C52	−112.76 (18)
N1—C20—C21—C22	65.0 (2)	N2—C46—C47—C48	−59.5 (2)
C18—C20—C21—C22	−61.1 (2)	C44—C46—C47—C48	65.9 (2)
C26—C21—C22—C23	2.2 (3)	C52—C47—C48—C49	−0.8 (3)
C20—C21—C22—C23	−176.92 (15)	C46—C47—C48—C49	−179.45 (16)
C21—C22—C23—C24	−0.4 (3)	C47—C48—C49—C50	0.7 (3)
C22—C23—C24—C25	−1.6 (3)	C48—C49—C50—C51	−0.2 (3)
C23—C24—C25—C26	1.8 (3)	C49—C50—C51—C52	−0.2 (3)
C24—C25—C26—C21	0.0 (3)	C48—C47—C52—C51	0.4 (3)
C22—C21—C26—C25	−2.0 (3)	C46—C47—C52—C51	179.09 (16)
C20—C21—C26—C25	177.13 (16)	C50—C51—C52—C47	0.0 (3)

### Hydrogen-bond geometry (Å, º)

Cg1 is the centroid of the C35–C40 ring.

**Table:**

*D*—H···*A*	*D*—H	H···*A*	*D*···*A*	*D*—H···*A*
C29—H29···Cl1	0.95	2.73	3.678 (2)	174
C24—H24···*Cg*1^i^	0.95	2.95	3.680 (2)	135

Symmetry code: (i) *x*+1, −*y*−1/2, *z*−1/2.

## References

[bb1] Agilent (2012). *CrysAlis PRO* Agilent Technologies, Yarnton, England.

[bb2] Brandenburg, K. (2006). *DIAMOND* Crystal Impact GbR, Bonn, Germany.

[bb3] Farrugia, L. J. (1997). *J. Appl. Cryst.* **30**, 565.

[bb4] Gans, J. & Shalloway, D. (2001). *J. Mol. Graph. Model* **19**, 557–559. 10.1016/s1093-3263(01)00090-0 11552684

[bb5] Ramachandran, R., Rani, M., Senthan, S., Jeong, Y.-T. & Kabilan, S. (2011). *Eur. J. Med. Chem.* **46**, 1926–1934. 10.1016/j.ejmech.2011.02.036 21397368

[bb6] Ramalingan, C., Balasubramanian, S., Kabilan, S. & Vasudevan, M. (2004). *Eur. J. Med. Chem.* **39**, 527–533. 10.1016/j.ejmech.2004.02.005 15183911

[bb7] Ramalingan, C., Ng, S. E. & Tiekink, E. R. T. (2012). *Acta Cryst* E**68**, o2300. 10.1107/S1600536812029200 PMC3394076 22798941

[bb8] Sheldrick, G. M. (2008). *Acta Cryst.* A**64**, 112–122. 10.1107/S0108767307043930 18156677

[bb9] Spek, A. L. (2009). *Acta Cryst.* D**65**, 148–155. 10.1107/S090744490804362X PMC2631630 19171970

[bb10] Westrip, S. P. (2010). *J. Appl. Cryst.* **43**, 920–925.

